# Silica Particles Mediate Phenotypic and Functional Alteration of Dendritic Cells and Induce Th2 Cell Polarization

**DOI:** 10.3389/fimmu.2019.00787

**Published:** 2019-04-24

**Authors:** Suna Liu, Changfu Hao, Lei Bao, Dehua Zhao, Hongyi Zhang, Jianyong Hou, Di Wang, Huiting Chen, Feifei Feng, Wu Yao

**Affiliations:** ^1^Department of Henan Newborn Screening Center, Department of Pediatrics, Third Affiliated Hospital of Zhengzhou University, Zhengzhou, China; ^2^Department of Public Health, Zhengzhou University, Zhengzhou, China; ^3^Hospital Infection Management, The First Affiliated Hospital of Henan University, Kaifeng, China

**Keywords:** dendritic cells, silica, silicosis, alveolar macrophages, phagocytosis, Th2 polarization

## Abstract

During silicosis, immune cells, including macrophages, T cells, B cells, and NK cells, participate in fibrosis development through alteration of the immune status. Dendritic cells (DCs) are professional antigen-presenting cells (APCs) with a key role in initiating immune responses and sustaining immune tolerance to maintain homeostasis. The relative contribution of DCs to silicosis progression is not well-documented. In the current study, we investigated the phenotypic and functional alterations of peripheral blood mononuclear cell (PBMC)-derived DCs of Sprague–Dawley (SD) rat during immune responses to silica exposure. We established models for direct and indirect exposure of DCs to silica by either treating DCs with silica or coculturing them with alveolar macrophages (AMs) treated with silica, respectively. The functional activity of DCs was analyzed by measuring their expression of costimulatory molecules, fluorescent microparticle uptake, cytokine production, and ability to mediate T cell polarization *in vitro*. *In vivo*, we demonstrated that silica could induce DC migration in response to silica exposure. Our results show that cytokine production by DCs was increased in response to direct silica direct exposure, while indirect silica exposure led to reduced cytokine levels. Moreover, the phagocytic capacity of DCs increased in cocultures after silica exposure. Gene and protein expression analyses showed that silica exposure altered the expression levels of Toll-like receptor pathway proteins and inflammatory factors. DC surface expression of the costimulatory molecules, CD80, CD86, and major histocompatibility complex, was inhibited by exposure to silica, which mediated a Th2-polarizing response *in vitro*. In rats, silica exposure induced migration of DCs from the peripheral blood into the alveoli. These results demonstrate that direct and indirect exposure to silica particles alter the phenotype and function of DCs, thereby regulating immune responses. Such changes may contribute to the development of silicosis by altering DC phenotype, function, and migration and by influencing the balance between Th1 and Th2 cells.

## Introduction

Silica particles are the typical environmental and occupational cause of silicosis (1). The development of silicosis is a complex process associated with the activation of inflammatory cells, creation of inflammatory mediators, and initiation of adaptive immune response; however, the majority of past investigations of silicosis appear to have overlooked the importance of dendritic cells (DCs). DCs are critical antigen-presenting cells (APCs) that contribute to immune response initiation (2–4) and are important regulators of T cell activity (5, 6). Depending on their ontogeny and state of differentiation and maturation, DCs can initiate immune responses and also induce immune tolerance as well as determine the direction of Th1/Th2 polarization (7, 8). Although silica particles directly alter the cellular and molecular biological characteristics of immunocompetent cells, in response to chronic and recurrent encounters in the lung (9), their ability to affect DCs is not well-studied. Therefore, a systemic study of the roles of DC in silicosis is required.

Alveolar macrophages (AMs) are generally considered the primary phagocytes involved in responses to the inhalation of silica. AMs are critical effector cells and the first defense against foreign substances in pneumoconiosis. They phagocytose inhaled silica and become activated, releasing various inflammatory cytokines that initiate immune responses to foreign substances (10–12). DCs have a key role in the control of lung immune responses (13), and a recent report found that DCs can phagocytose mesoporous silica particles (14). Interactions between different types of lung cells are crucial for the outcome of alveolar epithelial repair (15). The lung hosts AMs and DCs as its predominant phagocytic populations in both steady-state and disease conditions (16). AMs and DCs localize close to one another, and AMs release factors that suppress adaptive immunity by influencing the antigen-presenting capacity and activation of pulmonary DCs (17, 18). AMs and DCs are thought to play opposing roles during the development and maintenance of inflammation (19). During the early lung inflammation in silicosis, both the percentage and absolute number of AMs decrease significantly over time, with a concomitant significant increase in DCs, suggesting that DC-mediated immune activation may have a crucial role in the inflammation associated with silicosis (20). DCs can recognize antigens, present them to T or B cells, and induce a series of immune responses; however, whether DCs can recognize and phagocytose silica particles remains unclear. To address this question, we must first observe the consequences of direct silica contact with DCs, acting as either phagocytic cells or APCs, *in vitro*. In addition to responding directly to silica exposure, it is conceivable that silica may influence DCs indirectly via the influence of AMs activated in response to silica, which could alter the function of nearby DCs to regulate T cell activity. Hence, the development of *in vitro* experimental models of direct and indirect DC exposure would be desirable to facilitate analysis of the potential impact of silica on DCs.

In the present study, we aimed to examine the potential impact of silica on DCs. First, uptake of fluorescent microparticles and silica particles by DCs was analyzed by flow cytometry and electron microscopy, respectively, to assess the phagocytic capacity and pattern of DC phagocytosis of silica particles. Additionally, we examined the potential of silica to induce the release of inflammatory chemokines by ELISA analysis. The expression levels of IL-12, IL-18, TLR4, TLR9, Myd88, and NF-κB were determined by Western blotting and qPCR, while phenotypic changes in DC and T cell responses were detected by flow cytometry of coculture models. Furthermore, we evaluated the migration of DCs during immune responses to silica *in vivo*. Importantly, this research addresses current limitations of the understanding of DC functional alterations in response to silica exposure. We also collected direct evidence of DCs phagocytizing silica particles and the possible patterns and mechanisms involved in this process.

## Materials and Methods

### Animals

Sprague–Dawley (SD) rats (5- to 7-week-old males) were purchased from Henan Province Animal Center (Henan, China). All animal procedures were approved by the ethics committee of Zhengzhou University (Henan, China) and performed in accordance with the Guiding Principles for the Care and Use of Laboratory Animals. All research protocols were approved by the Animal Ethical Committee of Zhengzhou University (SYXK2016-0035).

### Generation of Immature DCs

DCs were obtained from rat peripheral blood mononuclear cells, isolated by density gradient centrifugation on Ficoll-Paque (Solabo, Beijing, China). Cells were cultured in RPMI 1640 medium supplemented with 15% heat-inactivated fetal bovine serum (FBS) in the presence of 5 ng/mL GM-CSF (518-GM-025) and 5 ng/mL interleukin-4 (504-RL-025). TNF-α (510-RT-010) (10 ng/mL) was added on day 5 to generate DCs. Cell factors were added once every 3 days, and the concentrations of cell factors remained unchanged. Cultures were maintained at 37°C in a 5% CO_2_ humidified atmosphere for 12 days before use in further experiments. All cytokines were purchased from R&D Systems, Minneapolis, MN, USA.

### Bronchoalveolar Lavage

Rats were anesthetized with 5% pentobarbital by intraperitoneal injection. After tracheal exposure and cannulation, bronchoalveolar lavage (BAL) was performed by infusing the lungs five times with 6 ml of sterile 0.9% saline. Bronchoalveolar lavage fluid (BALF) was collected and centrifuged (250 × g, 4°C, 10 min) to pellet AMs, which were resuspended in RPMI 1640. The number of AMs was counted using a hemocytometer (Invitrogen, Carlsbad, CA, USA), and the cells were incubated in 5% CO_2_ at 37°C for 2 h. The culture medium was then changed, and non-adherent cells were washed away; adherent cells were cultured in RPMI 1640 containing 15% FBS in 5% CO_2_ at 37°C.

### Toxicity Assay

DCs and AMs (1.0 × 10^4^/well) were plated in 96-well plates (three wells per group) and treated with silica (0–140 μg/ml) for 12, 24, and 48 h. CCK8 reagent (10 μl) (Dojindo, Kumamoto, Japan) was added to each well, cells were incubated for 1 h at 37°C, and the cytotoxicity of silica was measured by determining absorption at 450 nm using a microplate reader (Tecan Infinite M200; Tecan, Wetzlar, Germany), according to the manufacturer's instructions. In experimental settings, 48 h of exposure was used, which was predetermined by functional analysis after different exposure time (Supplementary Figure 1).

### Particle Exposure and Transwell Cocultures

Crystalline silica was used at 80 μg/ml, as this induced significant functional alterations of DCs. DCs were cultured in monocultures or cocultures. For cocultures of AMs and DCs, 0.5 × 10^6^ AMs were plated onto the upper side of Transwell inserts (0.4-μm pore size polyester membranes precoated with poly-L-lysine; Corning, NY, USA). Transwells were positioned ~2 mm above DC culture plates, with the AMs grown on the Transwells separated from DCs by the permeable Transwell membrane. Silica (median diameter, 1–5 μm; Sigma-Aldrich, St. Louis, USA) was added to DCs in monocultures or to AMs in cocultures. Controls consisted of DCs cultured without silica. After 48 h of silica treatment, cell culture supernatants and cells were collected for subsequent experiments.

### Isolation of T Cells and *in vitro* System for Coculture of T Cells and DCs

Rat splenic T cells were prepared by filtration through a nylon wool column. Before use, columns were equilibrated by washing with 20 ml RPMI 1640 and were incubated for 30 min in 5% CO_2_ at 37°C. Rat spleen cells were washed with Hanks' balanced salt solution. After lysis of red blood cells using RBC lysis buffer (BD Pharmingen, Franklin Lakes, NJ, USA), cells (2 × 10^8^) subjected to nylon wool purification were resuspended in 2 ml of warm RPMI 1640, loaded onto the column, and washed with 2 ml warm RPMI 1640. The column was sealed and incubated at 37°C, 5% CO_2_ for 45 min. Non-adherent cells were eluted with 10 ml warm RPMI 1640 (37°C). T cell purity was 94.6% as determined by flow cytometry. Eluted cells were collected by centrifugation and passed through a second nylon wool column. T cells were washed twice and then T cells were cocultured with silica-conditioned DCs at a ratio of 10:1. The positive control group were set up to ensure stainings for IFN-γ and IL-4 in optimal conditions, while in the positive control group, T cells were monocultured and stimulated with 200 U/ml IL-12 and 10 μg/ml anti-IL-4 for Th1, and 10 μg/ml IL-4 for Th2 (Supplementary Figure 2). After 24 h, cocultured cells were visualized by phase-contrast microscopy, the coculture supernatant was collected for detection of cytokines, and proportions of Th1 and Th2 cells were detected by flow cytometry.

### Cytokine Assay

Cytokine levels in coculture supernatants were measured using commercially available kits for rat IL-12p70, IL-18, IL-4, and IFN-γ (eBioscience, San Diego, CA, USA), as specified by the manufacturers. The lower detection limits were 3.5 pg/ml for IL-12p70, 18 pg/ml for IL-18, 0.2 pg/ml for IL-4, and 2 pg/ml for IFN-γ. Assays were repeated twice, and three samples were collected for each assay.

### Flow Cytometry Analysis

For DC phenotype analysis, DCs were stained with the following antibodies: FITC-conjugated CD86, PE-conjugated CD83, and PE-conjugated class II major histocompatibility complex (MHC-II) (all from BD Biosciences, San Jose, CA, USA). Corresponding isotype-matched antibodies were used as negative controls. The FACSVerse instrument and FACS Suite software (Accuri C6; BD Biosciences, Franklin Lakes, NJ, USA) were used to acquire data. Results are presented as the percentage of positive cells within a given population, defined using the geometrical mean fluorescence intensity (MFI). Analysis was conducted using the flow cytometer software (BD Biosciences).

Following coculture with DC, T cells were stained for surface and intracellular markers as previously described (21). Cells were incubated with phorbol myristate acetate (50 ng/ml; Sigma-Aldrich, St. Louis, USA) and ionomycin (800 ng/ml; Sigma-Aldrich, St. Louis, USA) for 5 h. Monensin (2 μM; BD Biosciences, San Diego, CA, USA) was also added for the final 2 h of activation as a protein transport inhibitor. For surface staining, T cells were stained using PerCP-conjugated CD3 and FITC-conjugated CD4 antibodies (BD Biosciences). Cells were washed, fixed, and then permeabilized using a Cytofix/Cytoperm kit (BD Pharmingen, Franklin Lakes, NJ, USA) according to the manufacturer's instructions. Thereafter, cells were intracellularly stained with Alexa Fluor 647-conjugated IFN-γ antibody to identify Th1 cells and PE-conjugated IL-4 antibody to identify Th2 cells. Corresponding isotype-matched antibodies were used as negative controls. Detailed gating strategies can be found in Supplementary Figure 2A in the Supplementary Material. Dead cells were gated out depending on forward scattering and side scattering.

Phagocytosis was assessed by overlaying monolayers with carboxylated yellow-green fluorescent polystyrene microparticles (FMs; 2 μm, Invitrogen, Carlsbad, CA, USA). The microparticles were opsonized in 3% bovine serum albumin (Sigma-Aldrich, St. Louis, MO, USA) at room temperature for 30 min. Cells and microparticles were gently mixed in RPMI 1640 and incubated at 37°C in 5% CO_2_ for 30 min. Phagocytosis of fluorescence-labeled beads by cells was analyzed by flow cytometry (Accuri C6; BD Biosciences, Franklin Lakes, NJ, USA). Cell-phagocytosed FMs were visualized by phase-contrast and fluorescence microscopy (Eclipse TS100-F; Nikon, Tokyo, Japan).

To track the ratio of carboxyfluorescein diacetate succinimidyl-ester (CFSE)-labeled DCs to DCs after silica exposure, the numbers of DCs in alveolar lavage fluid from SD rats were determined using DC-specific markers (OX-62) by flow cytometry. DCs were stained with Alexa Fluor 647-conjugated OX62 (BD Biosciences, San Jose, CA, USA). Corresponding isotype-matched antibody was used as negative control. Cells gated on Alexa Fluor 647-conjugated OX62 were separated based on CFSE vs. side scatter (SSC). The FACS analysis was performed as mentioned above.

### Scanning Electron Microscopy and Transmission Electron Microscopy

For scanning electron microscopy (SEM) analysis, DCs were fixed in 1% glutaraldehyde (4°C, phosphate buffer, pH 7.4), and then rinsed in PBS to remove the fixative. Fixed cells were dehydrated in a graded ethanol series (70, 80, 90, 95, then 100%), critical point dried from liquid CO_2_, and viewed in a scanning electron microscope (S-3500N, Hitachi, Tokyo, Japan).

For transmission electron microscopy (TEM) analysis, DCs were fixed, washed, and then dehydrated in a graded ethanol series, as described above. Ethanol was gradually replaced with propylene oxide before infiltration and embedded in epoxy resin. Ultrathin sections were cut using an ultramicrotome (Reichert, Vienna, Austria) and collected on 200-mesh carbon-coated copper grids. After staining with uranyl acetate and counterstaining with lead citrate, samples were observed by TEM using a Hitachi H-7500 (Hitachi, Tokyo, Japan).

### Gene Expression Analysis

RNA was extracted from DCs in different cultures using Trizol reagent (Invitrogen, USA). A PrimerScript RT reagent kit (Takara, Dalian, China) and SYBR-Green II (Takara, Dalian, China) were used according to the manufacturer's instructions. Quantitative real-time PCR assays were run and analyzed using an ABI7500 PCR system. Primer sequences were as follows: *IL-12* sense 5′-AAGGTCACACTGAACCAAAGG-3′ and *IL-12* antisense 5′-TGATGTCCCTGATGAAGAAGC-3′; *IL-18* sense 5′-TCAGACCACTTTGGCAGACTT-3′, and *IL-18* antisense 5′-TAGGGTCACAGCCAGTCCTC-3′; *TLR4* sense 5′-CTCACAACTTCAGTGGCTGGATTTA-3′ and *TLR4* antisense 5′-GTCTCCACAGCCACCAGATTCTC-3′; *TLR9* sense 5′-GGCCACAGGACTCAAGAGCA-3′ and *TLR9* antisense 5′-AGAGGCCTATCACAGCCATCAAG-3′; *Myd88* sense 5′-TATACCAACCCTTGCACCAAGTC-3′ and *Myd88* antisense 5′-TCAGGCTCCAAGTCAGCTCATC-3′; *NF*κ*B* sense 5′-TCTTCGACTACGCGGTTACGG-3′ and *NF*κ*B* antisense 5′-CTCACGAGCTGAGCATGAAGG-3′; and *GAPDH* sense 5′-GGCACAGTCAAGGCTGAGAATG-3′ and *GAPDH* antisense 5′-ATGGTGGTGAAGACGCCAGTA-3′.

### Western Blot Analysis

Cells were lysed in RIPA Lysis Buffer (Beyotime, Haimen, China). Lysates were then separated on a 10% SDS polyacrylamide gels and transferred onto polyvinylidene difluoride membranes (Millipore). Membranes were blocked with 5% milk for 1 h and incubated overnight at 4°C with primary antibodies. Primary antibodies, including anti-IL12A antibody (1:1,000, ab133751), anti-IL18 antibody (1:2,000; ab191860), anti-NF-κB p50 (1:3,000, ab32360), and anti-GAPDH (1:500, ab22048), were purchased from Abcam (Cambridge, MA, USA). After rinsing, immunocomplexes were incubated with horseradish peroxidase-conjugated anti-rabbit or anti-mouse IgG (1:2,000, Zhongshanjinqiao, Beijing, China) for 30 min at 37°C. Immunoreactive bands were visualized using an enhanced chemiluminescence system (ECL; GE Healthcare, Madrid, Spain).

### Animal Experiments

Twelve male rats were used for experiments and divided randomly into two experimental groups: control (*n* = 6) and silicosis (*n* = 6). Rats in the silicosis group were anesthetized using ether and received a single intratracheal instillation of silica suspension (0.5 ml; equivalent to 50 mg silica dust). Control animals were treated in the same way but received 0.5 ml sterilized saline. For CFSE labeling, DCs (2 × 10^6^ per mL) were incubated at 37°C for 30 min in 5 mM CFSE (Molecular Probes, Eugene, OR), washed, and transferred to silica and control group recipients via tail vein injection, following intratracheal instillation. After 8 days, BAL was collected for further analysis.

### Fluorescence Microscopy

After alveolar lavage, cells were resuspended in RPMI 1640 supplemented with 15% heat-inactivated FBS and then allowed to adhere 24-well-plates for 2 h. Non-adherent cells were removed by gentle aspiration, and adherent cells were cultured in RPMI 1640 medium supplemented with 15% heat-inactivated FBS. Cells were examined using light and polarizing microscopy (Eclipse TS100-F; Nikon, Tokyo, Japan).

### Statistical Analysis

Data are expressed as the mean ± SD. Statistical analyses were performed using one-way analysis of variance with appropriate least significant difference *post hoc* tests to compare experimental groups in GraphPad Prism 5.0 software. *p* < 0.05 were considered statistically significant. Statistical analyses of results are reported in the figure legends.

## Results

### Silica Induces Time- and Concentration-Dependent Cell Death

**Figure 1:**
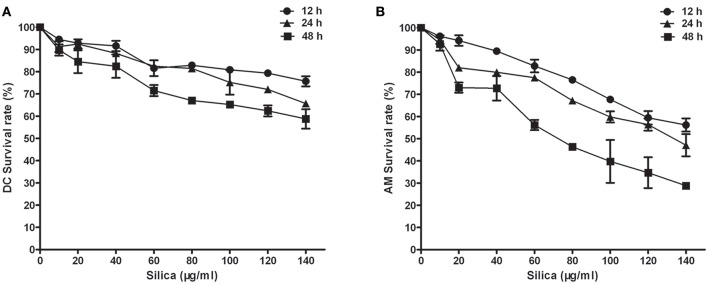
The effect of silica on cell viability. Survival curves of dendritic cells (DCs) **(A)** and alveolar macrophages (AMs) **(B)**. Silica-induced cell death was time and concentration dependent. Cell viability was examined by CCK8 assay using rat monocyte-derived DCs and AMs incubated with various concentrations of silica (0–140 μg/ml) for 12, 24, and 48 h. *n* = 6. Data are shown as mean ± SD.

To assess the effects of indirect exposure to silica on DCs via AMs, we first determined the toxicity of silica to DCs and AMs. DCs and AMs were exposed to varying concentrations (0–140 μg/ml) of silica to determine the lowest concentration at which particles were toxic to cells. Cell viability was examined after 12, 24, and 48 h using the CCK8 assay. Concentrations of silica as low as 80 μg/ml killed 32.96% of DCs, and 53.64% of AMs by 48 h. Exposure to silica significantly and dose- and time-dependently decreased the viability of AMs (Figure 1B), whereas exposure of DCs resulted in inhibition of their proliferation, rather than inducing cell death (Figure 1A). To study the direct and indirect impact of silica on DCs in the same exposure environment, DCs and AMs were exposed to the same and lower concentrations of silica, to ensure the functional activity of AMs. Based on these results, a concentration of 80 μg/ml of silica was used for all subsequent experiments.

### Dendritic Cell Phagocytic Activity

**Figure 2:**
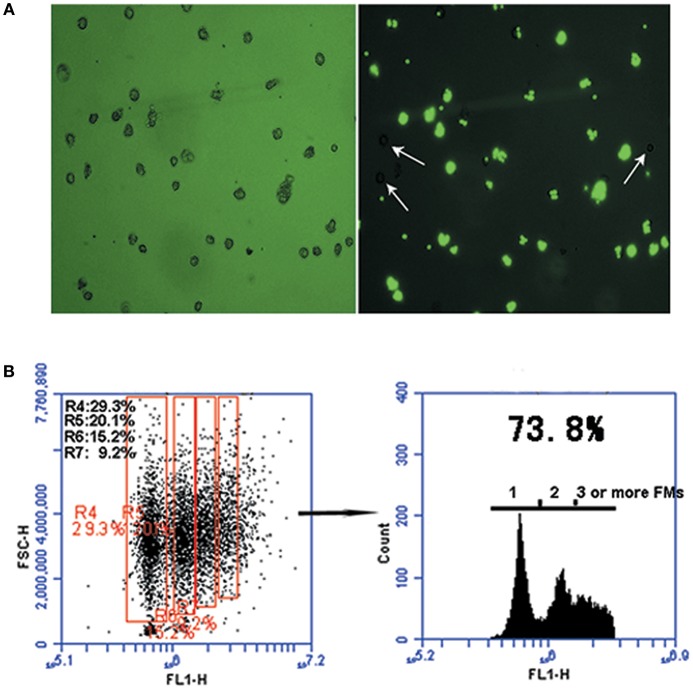
The phagocytic function of monocyte-derived DCs. **(A)** Representative micrographs of carboxylated yellow-green fluorescent polystyrene microparticles (FMs) (green) phagocytized by DCs. Cells were processed 30 min after phagocytosis. Light microscopic analysis showing FMs phagocytosed by DCs (left panel) and DCs that did not phagocytosed FMs (right panel, white arrow-heads); image from fluorescence microscopy showing FMs phagocytosed by DCs (right panel). **(B)** Graph representative of flow cytometry data illustrating DC phagocytosis of FMs. Numbers in the red boxes represent the percentages of cells that phagocytosed different numbers of FMs.

Phagocytic activity was observed in DCs isolated from peripheral blood monocytes by flow cytometry analysis. DC phagocytosis of FMs was visualized using phase-contrast and fluorescence microscopy. The phagocytic capacity of cells was determined based on the proportions containing one, two, three, four, or more beads (scatter plots, Figure 2B). After 30 min of exposure to FMs, cells containing no beads (white arrow-heads) and containing beads varied in size, morphology, and numbers of beads ingested, as determined by fluorescence microscopy (Figure 2A). The overall proportion of cells containing FMs reached 73.8%, with 29.3% containing one, 20.1% two, 15.2% three, and 9.2% four or more FMs.

### Dendritic Cell Phagocytosis of Silica

**Figure 3:**
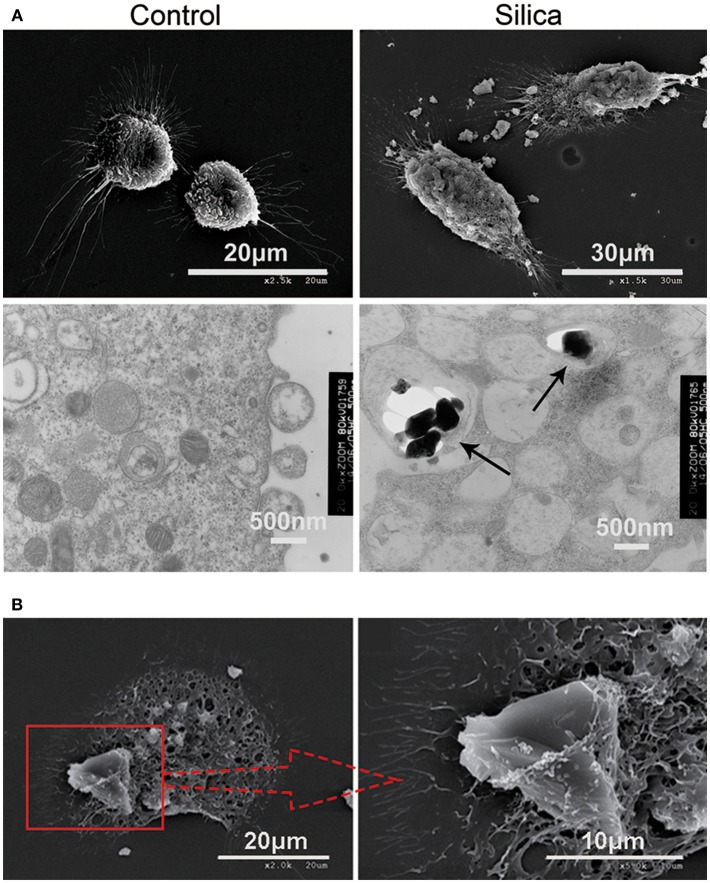
Representative scanning electron microscopy (SEM) and transmission electron microscopy (TEM) images showing rat monocyte-derived DCs phagocytosing silica. **(A)** SEM showing that the surface of untreated cells displays prominent ruffles and dendritic pseudopodia. Cells treated with silica appeared larger with marked tumescence, and the ruffles on the cell surface appear shallower, or absent. In addition, numerous large voids are visible on the cell surface. TEM showing untreated cells containing an abundance of organelles and normal vacuoles, whereas numerous large vacuoles and few organelles were visible in the cell cytoplasm of cells treated with silica. Silica is visible inside vacuoles in DCs (black arrow-heads). **(B)** SEM showing a rat monocyte-derived DC phagocytosing a silica particle.

To further investigate whether DCs can directly phagocytose silica, we treated them with silica (80 μg/ml) for 48 h, and then examined their morphology and structure by SEM and TEM. Untreated DCs (control) exhibited prominent surface ruffles and dendritic pseudopodia, while SEM revealed normal cell ultrastructure. Cells treated with silica appeared larger and markedly swollen, surface ruffles were shallower or absent, and the surface membranes contained crater-like cups. TEM analysis showed that untreated DCs had abundant organelles and frequently contained lysosomes. Larger and markedly swollen vacuoles were visible in silica-treated DCs, with silica particles inside the vacuoles (Figure 3A). The SEM showed that DCs phagocytosed large silica particles by endocytosis, and the surface membrane of silica-treated DCs appeared vacuolated (Figure 3B). The SEM observation of the interiors of cells treated with silica revealed major changes, with numerous large vacuoles and decreased visible organelles in the cytoplasm. These findings indicate that DCs can phagocytose silica and produce numerous large vacuoles during this process.

### Morphological and Phenotypic Changes to Dendritic Cell Induced by Silica

**Figure 4:**
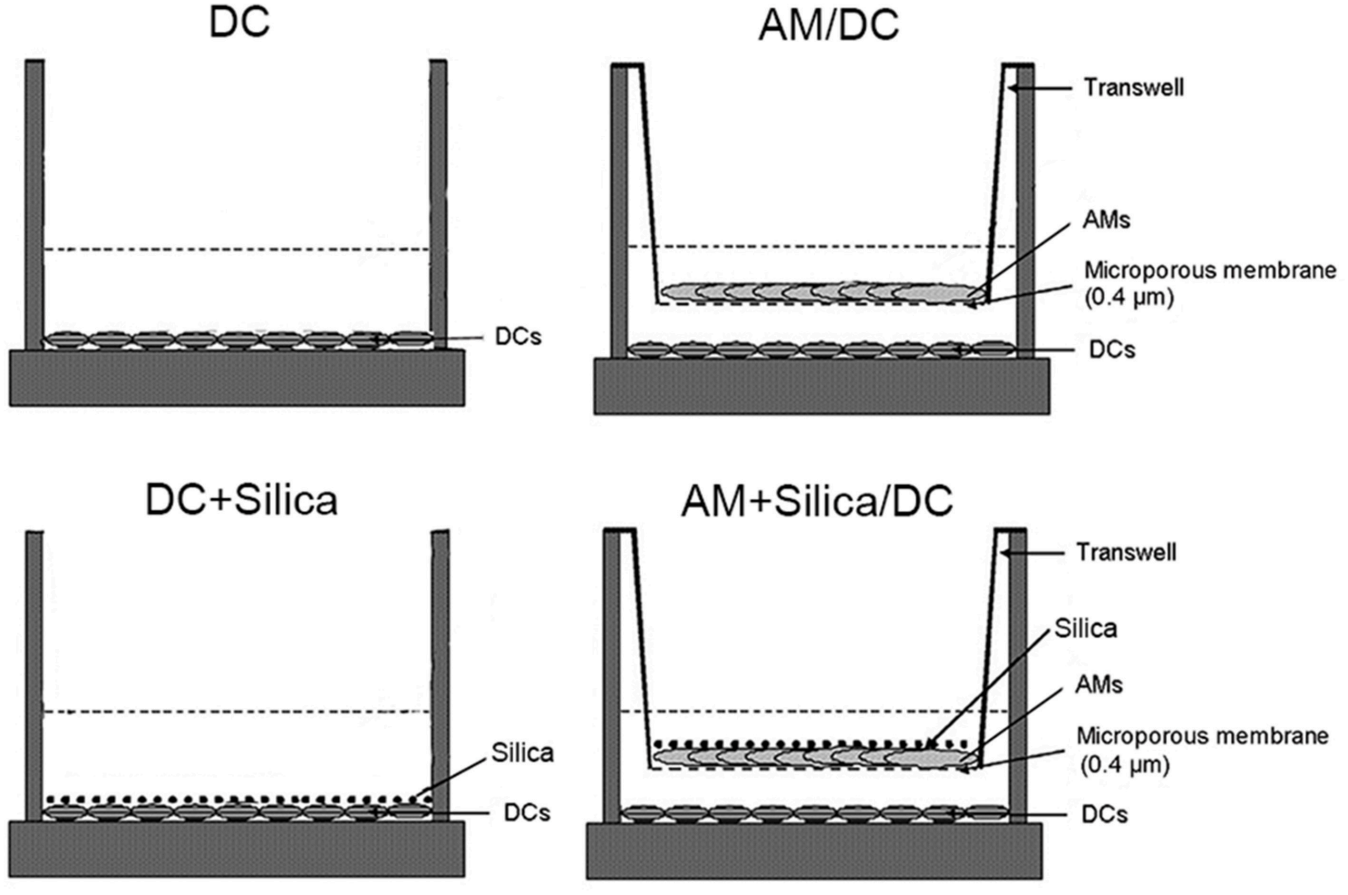
Experimental design for silica exposure of DCs in monocultures and cocultures with AMs.

The induction of phenotypic and functional changes in DC by exposure to silica was examined in monocultures of DCs directly exposed to silica (DC+silica), indirectly exposed by non-contact coculture with AMs (AM+Silica/DC), unexposed controls in non-contact coculture with AMs (AM/DC), and unexposed controls in monocultures of DCs (DC). For the experimental design, see Figure 4.

**Figure 5:**
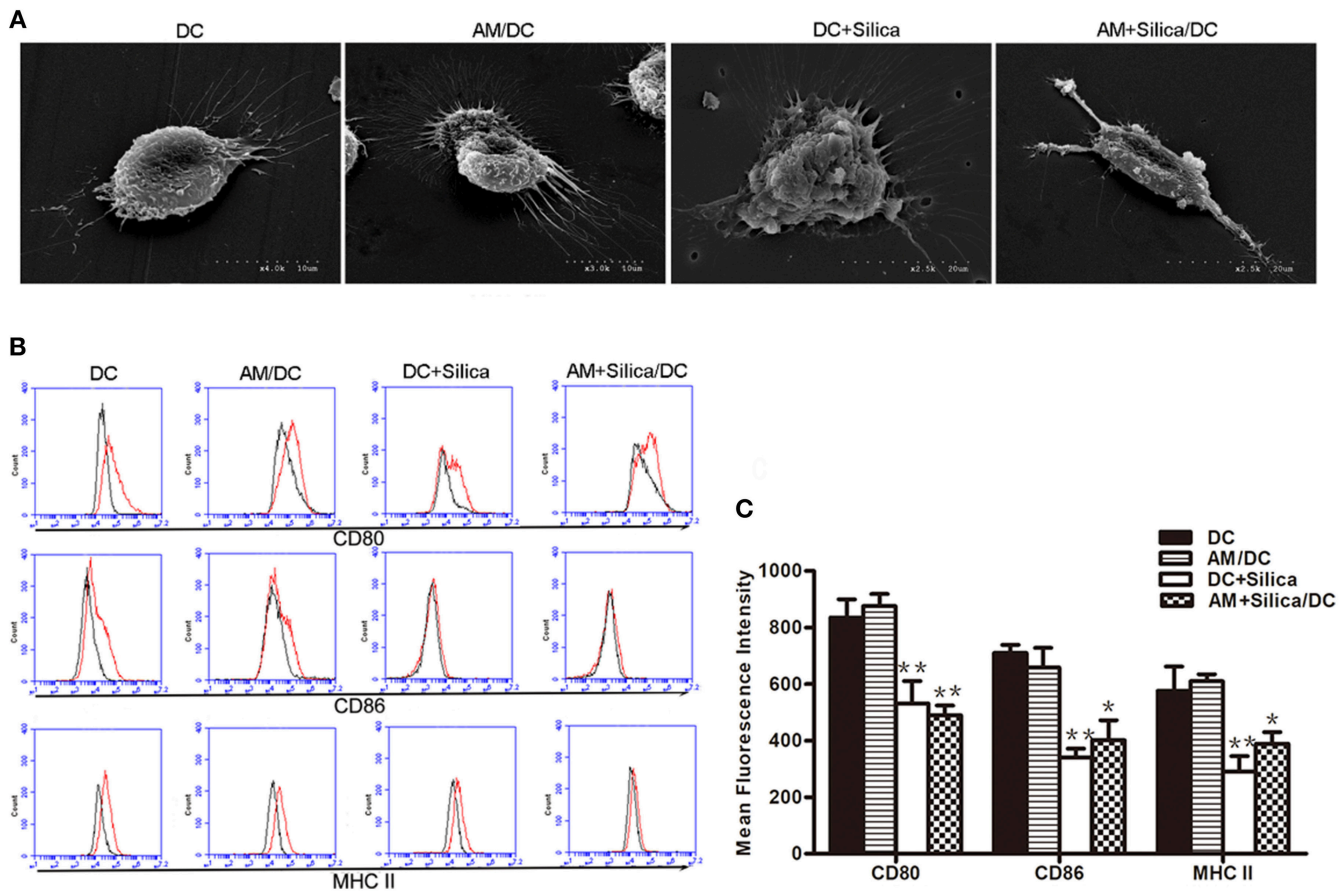
Silica alters the morphology of DCs and their expression of costimulatory molecules. **(A)** SEM images of rat monocyte-derived DCs under different culture conditions (DC, DC+Silica, AM/DC, and AM+Silica/DC). **(B)** Histogram and **(C)** representative data showing expression of surface markers CD80, CD86 and MHC-II as determined by flow cytometry on DCs under different culture conditions. Isotype control is shown as black line histogram. Live cells were selected prior to histograms, and levels of expression were compared to isotype controls. *n* = 3. *P < 0.05, **P < 0.01 compared with the DC control group. Results are the mean of three independent experiments (mean ± SD).

Immature DCs (iDCs) and mature DCs (mDCs) can be distinguished based on phenotypic and functional differences. When DCs undergo maturation, they express enhanced levels of antigen-presenting molecules (MHC-II) and costimulatory molecules (CD80 and CD86). To investigate the direct and indirect effects of silica particles on DC maturation, DCs were directly or indirectly exposed to silica particles for 48 h. Untreated DCs displayed round cell morphology; however, when treated with silica particles (DC+Silica group) they became larger and markedly swollen, while DCs cocultured with AMs treated with silica particles (AM+Silica/DC group) were primarily spindle shaped but did not exhibit swelling. In the AM/DC group, DCs had round cell morphology, similar to the control group (Figure 5A). As shown in Figures 5B,C, our data indicated that CD80, CD86, and MHC-II were expressed in DCs. Expression of these markers was inhibited by addition of silica particles in both the DC+Silica and AM+Silica/DC groups, relative to the control group. A slight decrease in levels of CD80, CD86, and MHC-II was detected in the AM/DC group; however, the difference was not statistically significant.

### Silica Alters the Phagocytic Capacity of Dendritic Cells

**Figure 6:**
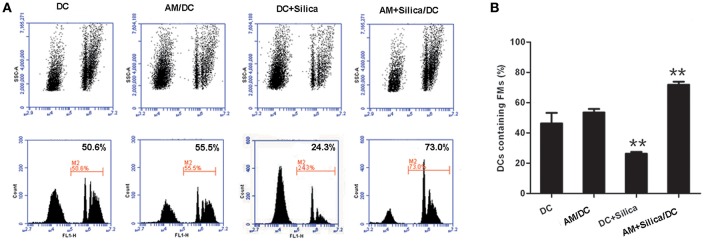
Silica alters the phagocytosis of DCs. **(A)** Representative flow cytometry data for DC phagocytosis of carboxylated yellow-green fluorescent polystyrene microparticles (FMs) under different culture conditions (DC, AM/DC, DC+Silica, and AM+Silica/DC). **(B)** Bar graphs showing the proportions of DCs that phagocytosed FMs. Phagocytosis experiments were conducted in RPMI 1640 medium containing 15% FBS at 37°C and 5% CO_2_ for 30 min. Data from 10,000 events were collected and plotted as fluorescence intensity vs. number of cells. Phagocytosis of FMs was evaluated by determining the percentage of FM-positive DCs in the total population of DCs. *n* = 5. **P < 0.01 compared with the non-silica-treated group. Results are the mean of three independent experiments (mean ± SD).

The morphological alterations we observed in DCs exposed to silica would certainly be expected to influence cell function, including antigen uptake, which is a functional characteristic of iDCs. To investigate the phagocytic ability of silica-exposed DCs, we used FMs. Our data demonstrated that DCs in the AM+Silica/DC group exhibited increased levels of microparticle phagocytosis, while levels were decreased in the DC+Silica group. There was no change in phagocytosis of microparticles in the DC in the AM/DC groups compared with controls (Figures 6A,B).

### Silica Alters the Release of IL-12p70 and IL-18 by Dendritic Cells

**Figure 7:**
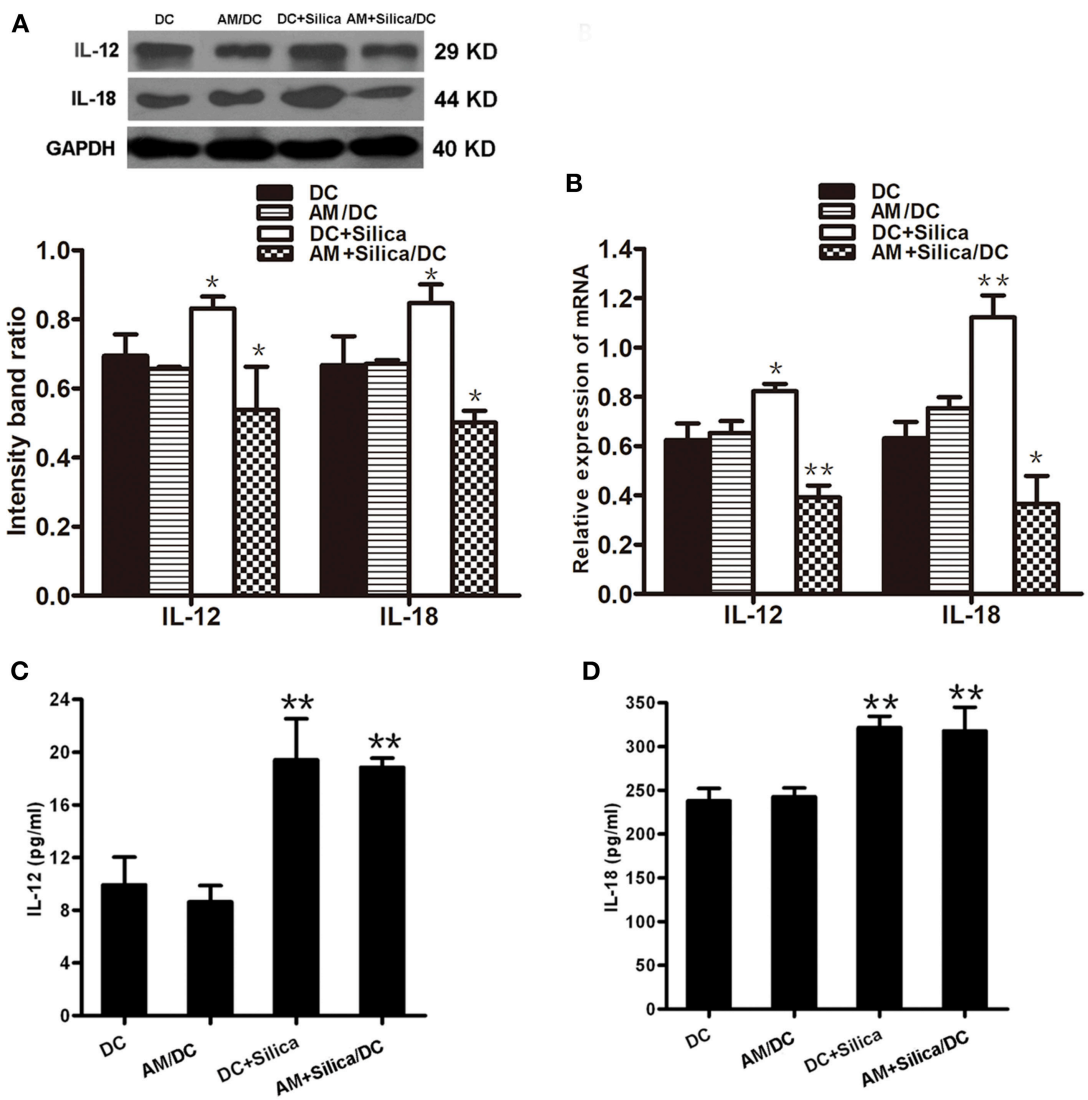
Direct or indirect silica exposure alters the expression of cytokines by DCs in culture. **(A)** IL-12 and IL-18 protein expression by DCs after 48 h of coculture with silica treated by Western blotting. Graph below shows statistical analysis of the results of immunoblotting. **(B)** *IL-12, IL-18*, and *NF-*κ*B* mRNA expression by DCs after 48 h of coculture with silica treated by real-time PCR. **(C,D)** ELISA showing the levels of IL-12 and IL-18 proteins in supernatants from DCs cocultured with AMs treated with silica (80 μg/ml, 48 h) compared with those in the non-silica-treated control group. Data are presented as mean ± SD from three independent experiments (*n* = 6). **(D)** Data represent at least three independent experiments. *P < 0.05; **P < 0.01 compared with the control group.

IL-12p70 and IL-18 are central factors to the adaptive immune response. To evaluate the impact of silica exposure on levels of *IL-12* and *IL-18* mRNA, DCs were grown in monocultures or cocultured with AM treated with silica (80 μg/ml) for 48 h, and the expression levels of *IL-12* and *IL-18* mRNA were determined by qPCR. Figure 7B shows that levels of *IL-12* and *IL-18* were significantly higher in the DC+Silica group than those in DC controls. *IL-12* and *IL-18* expression levels were also significantly downregulated in the AM+Silica/DC group compared with the DC control group. No distinct differences were observed between the AM/DC and DC control groups. Western blot assays generated results similar to those determined by qPCR (Figure 7A).

In addition, to confirm the effects of silica particles on inflammatory chemokine release by DCs exposed to silica (80 μg/ml) for 48 h, IL-12p70 and IL-18 levels in culture supernatants were measured using ELISA kits specific for each protein. Levels of IL-12p70 and IL-18 protein were quantified and normalized relative to the control group. As shown in Figures 7C,D, compared with controls, the production of IL-12p70 and IL-18 were significantly upregulated in the DC+Silica and AM+Silica/DC groups; however, there was no significant difference in the AM/DC group. These findings indicate that the presence of silica-exposed AMs contributed to the release of IL-12p70 and IL-18 in cocultures.

### Silica Alters the Expression of TLR4, TLR9, Myd88, and NF-κB in Dendritic Cells

**Figure 8:**
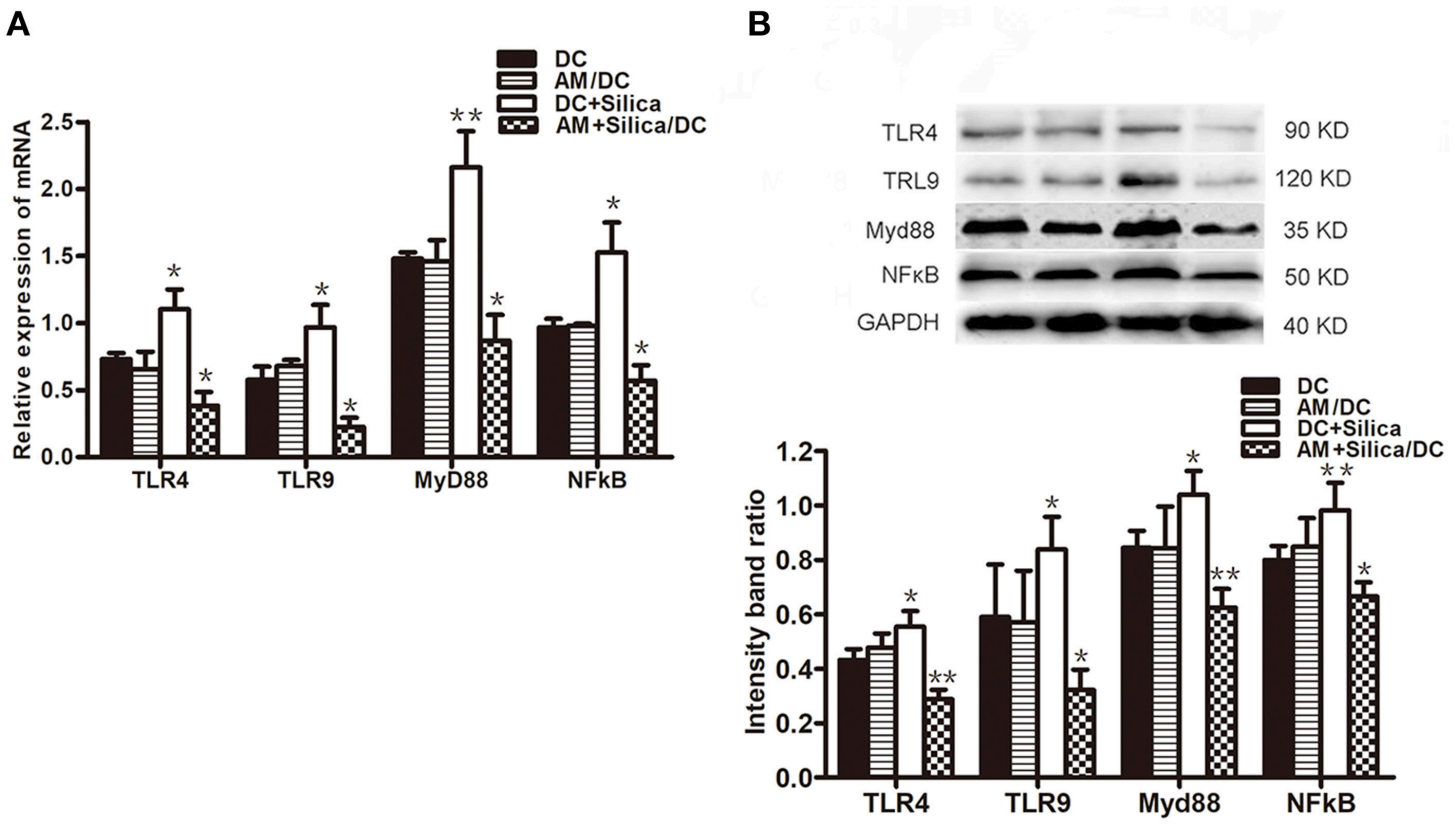
**(A)** *TLR4, TLR9, Myd88*, and *NF-*κ*B* mRNA expression levels in DCs after 48 h of coculture with silica treated by real-time PCR. **(B)** Graph of statistical analysis of immunoblots (above) of TLR4, TLR9, Myd88, and NF-κB p50 in DCs in various groups. *n* = 5. *P < 0.05; **P < 0.01 compared with the control group.

To evaluate the potential mechanism by which DCs phagocytose silica, the mRNA and protein levels of TLR4, TLR9, Myd88, and NF-κB in DCs exposed to silica were determined. As shown in Figure 8A, levels of *TLR4, TLR9, Myd88*, and *NF-*κ*B* mRNA were significantly upregulated in the DC+Silica group, compared with those in the control group, whereas levels were significantly downregulated in the AM+Silica/DC group, with no clear differences observed between the AM/DC and DC control groups. Western blotting assays demonstrated similar results to those of qPCR (Figure 8B). Taken together, our data demonstrate that silica can increase the expression levels of TLR4, TLR9, Myd88, and NF-κB in DCs by direct exposure; however, their levels were inhibited in response to indirect exposure.

### DC Exposure to Silica Induces Th2 Polarization

**Figure 9:**
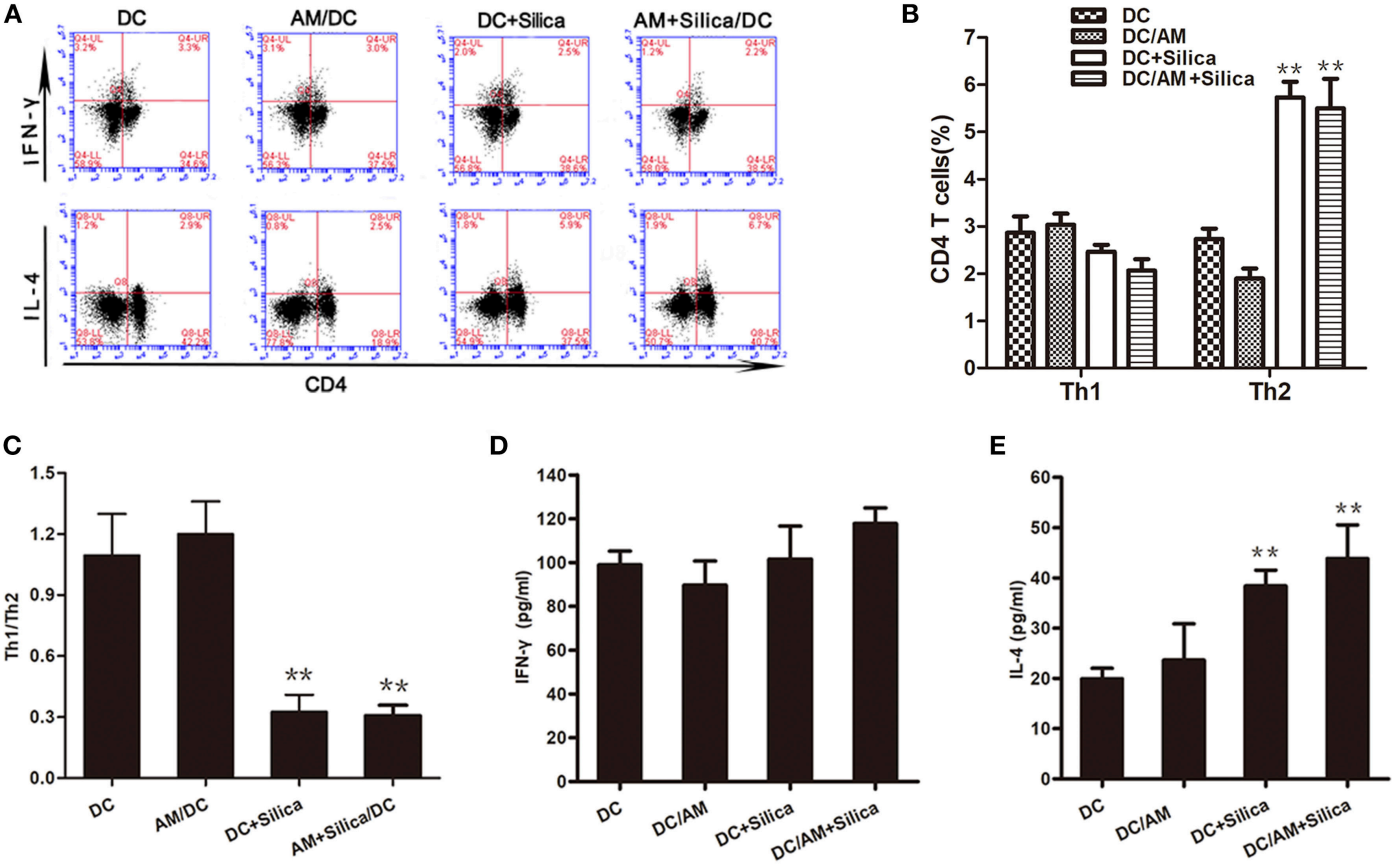
Exposure of DCs to silica induces Th2 polarization *in vitro*. **(A)** Flow cytometric detection of Th1 and Th2 cells in different cocultures. Stimulated T cells cocultured with DCs from different experimental groups (DC, DC+Silica, AM/DC, and AM+Silica/DC) for 24 h were stained with FITC-conjugated CD4 mAb and Alexa Fluor 647-conjugated interferon-γ mAb for Th1 cells, and PE-conjugated IL-4 mAb for Th2 cells. **(B)** Data from **(A)** presented as a bar graph of the proportions of CD4 T cells. **(C)** Data presented as a bar graph of the ratio of Th1 to Th2 cells. **(D,E)** ELISA showing the levels of IFN-γ and IL-4 proteins in supernatants from T cells cocultured with DCs treated with silica (80 μg/ml, 48 h) compared with those in the non-silica-treated control group. Data are presented as mean ± SD from three independent experiments (*n* = 6). **P < 0.01 compared with the control group. Results are the mean of three independent experiments (mean ± SD).

DCs have the unique ability to induce primary immune responses through activation and polarization of naive T cells. Therefore, we estimated the ratio of Th1 (IFN-γ^+^ CD4^+^) and Th2 (IL-4^+^ CD4^+^) cells among T cells cocultured with silica-exposed DCs by flow cytometry to evaluate the ability of DC exposure to silica to stimulate primary allogeneic T cell responses and influence the Th1/Th2 balance. Flow cytometric detection of Th1 and Th2 cells in the DC/AM, DC+Silica, AM+Silica/DC, and control groups indicated that coculture of T cells with DCs for 24 h significantly increased the proportion of Th2 cells in the DC+Silica and AM+Silica/DC groups compared with the DC control group, although there were no significant differences in the proportions of Th1 cells (Figures 9A,B); hence, the Th1/Th2 ratio was significantly decreased (Figure 9C). We therefore performed ELISAs to detect IFN-γ and IL-4 in coculture supernatants and found that Th1 cells and IFN-γ as well as Th2 cells and IL-4 had similar trends. Compared with the DC control group, IL-4 expression significantly increased in the DC+Silica and AM+Silica/DC groups (Figure 9E), although there were no significant differences in IFN-γ expression (Figure 9D). Based on these findings, it was apparent that DCs induce Th2 immunity in response to silica exposure.

### Silica Exposure Induces Dendritic Cell Migration *in vivo*

**Figure 10:**
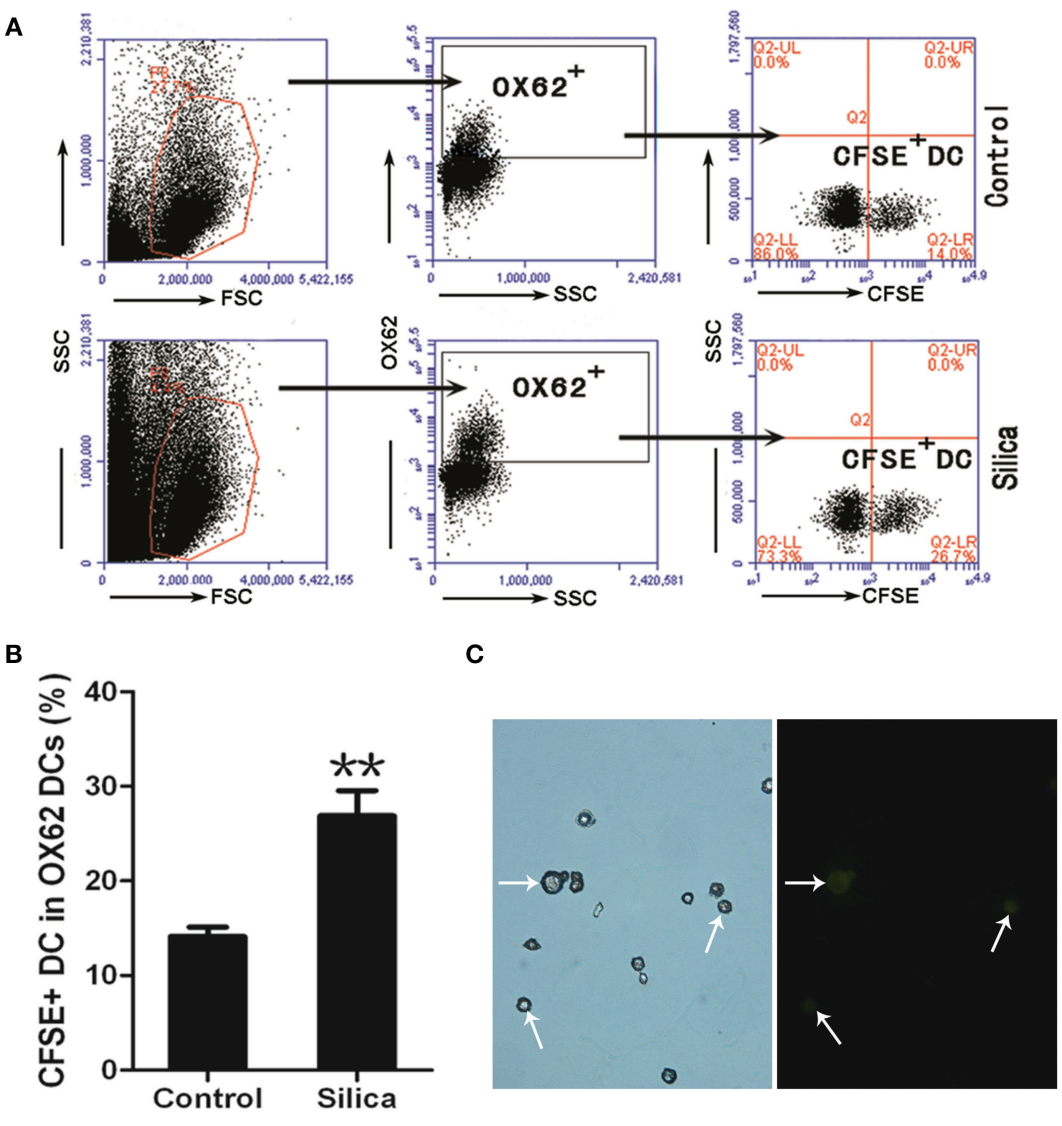
Silica promoted the migration of DCs. **(A)** Lung lavage cells from Sprague–Dawley (SD) rats exposed to saline or silica were examined by flow cytometry of the CFSE^+^ cell within the OX62^+^ DC population. **(B)** Bar graph showing the ratio of carboxyfluorescein diacetate succinimidyl-ester (CFSE)-labeled DC in OX62^+^ DC. The ratio of CFSE^−^ labeled DC to OX62^+^ DC was altered by silica, but not saline exposure (*n* = 6, in duplicate or triplicate). Percentage populations are indicated in each quadrant. FL1, green fluorescence, logarithmic scale. **(C)** Micrographs of CFSE-labeled DCs (whiter arrow-heads) in alveolar lavage fluid (400×). **P < 0.01 compared with the control group. Results are the mean of three independent experiments (mean ± SD).

To investigate whether silica particles impact the migration of DCs in pulmonary environments, we generated a rat model of acute lung injury by inducing intranasal instillation of crystalline silica particles. CFSE-labeled DCs were injected into rats in both the silica-treated and control groups via the tail vein. Cells isolated from the alveolar spaces were then examined using light and fluorescence microscopy and analyzed by flow cytometry to track the ratio of CFSE-labeled DCs to DCs 8 days after silica exposure. Cells gated on Alexa Fluor 647-conjugated OX62 were separated based on CFSE vs. side scatter (SSC; Figure 10A). The ratio of CFSE-labeled DCs was increased in the silica group compared with the control group (*p* < 0.01; Figure 10B). CFSE-labeled DCs were found in alveolar lavage fluid (Figure 10C). The ratio of CFSE-labeled DCs was increased in the silica-treated group compared with the control group. These results reveal migration of DCs to the lung in response to silica over time.

## Discussion

The present study provides evidence that direct or indirect silica exposure can modulate DC-mediated immune responses by influencing both the maturation and function of DCs *in vitro*. To investigate the direct effects of silica on DCs and its indirect effects on DCs via silica-exposed AMs, we first successfully generated rat DCs by culturing blood monocytes in the presence of GM-CSF, IL-4, and TNF-α for 12 days. Our study showed that DCs were adherent in our GM-CSF, IL-4, and TNF-α culture system, which is consistent with previous observations that the majority of DCs can adhere to plastic and that some even exhibit particularly strong adhesion characteristics (22).

The phagocytic activity of DCs is generally lower than that of more efficient phagocytes, such as AMs (23); however, we found that the DCs exhibited a high phagocytic uptake capacity. Our data demonstrate that silica can be phagocytosed by DCs and has low toxicity to DCs relative to AMs. DCs maintained their high phagocytic capacity, which may be attributable to the culture conditions or the nature of maturation stimulation provided by our approach. In addition, we evaluated the possible mechanism of phagocytosis. Our study showed that silica was present inside vacuoles in DCs and that silica-containing vacuoles did not fuse with lysosomes, exhibiting apparent disruption of the vacuole membrane. Moreover, our results showed that the biological changes induced by silica involved cell morphology and structure. These findings are consistent with earlier observations by Henderson et al. (24, 25). Our study provides evidence that silica particles are phagocytosed by internalization into DCs.

Silica exposure can lead to activation of the innate immune system, resulting in proinflammatory cytokine production (26). Recent evidence points to a prominent role for IL-18 and IL-12 in silica-induced inflammation and fibrosis (27, 28). In the current study, we observed significant inhibition of DC maturation in both direct and indirect exposure groups after 48 h, suggesting that soluble factors may contribute to the inhibitory mechanism. DC maturation arrest is clearly accompanied by low production of biologically active IL-12p70 secretion (29). Our data suggest that there was a robust decrease in the expression of IL-12p70 and IL-18 in DCs in the DC+Silica and AM+Silica/DC groups, compared with control DCs. Nevertheless, the levels of IL-12p70 and IL-18 in coculture supernatants increased in DC+Silica and AM+Silica/DC groups. Macrophages treated with silica secrete IL-18 and IL-12 (27, 30). Thus, we hypothesized that our results stem from indirect exposure to silica, because AMs produce IL-12p70 and IL-18 into the supernatant of cocultures in the AM+Silica/DC group; these findings deserve further investigation and suggest that the immunoregulatory ability of DCs is regulated by the induction of soluble factor secretion in response to silica.

The morphology and behavior of DCs alter in response to silica exposure, which could trigger phenotypic change (31). In addition to levels of MHC-II, changes in the expression of costimulatory molecules provide another important indicator of DC maturation (32). In the current study, short-term (48 h) silica exposure stimulated mature DCs toward a more immature cell phenotype, as revealed by the downregulation of CD80, CD86, and MHC-II, and alteration of phagocytic capacity. Data from flow cytometry analysis showed that the expression of CD80, CD86, and MHC-II on DCs was decreased in the DC+Silica and AM+Silica/DC groups, suggesting that some soluble factors may be secreted into the coculture supernatant that induce a blockade of DC maturation following both direct and indirect silica exposure. In addition to phenotype alterations, we observed a marked change in the phagocytic function of silica-exposed DCs. The phagocytic capacity of DCs decreased after silica stimulation in the DC+Silica group compared with control groups. Nevertheless, DCs in the AM+Silica/DC group exhibited greater phagocytic ability. Our data demonstrate that silica can be phagocytosed by DCs; therefore, we speculate that the difference in phagocytic capacity between the directly and indirectly exposed groups may be a consequence of direct silica exposure, because the phagocytic function of DCs had reached a saturation point after phagocytosing silica particles for 48 h, leading to a decrease in their phagocytic capacity; this hypothesis warrants further investigation. Thus, our data suggest that changes in phagocytic capacity may be related to a blockade of DC maturation following indirect exposure to silica.

Gene expression associated with the maturation and APC function of DCs is largely regulated by the transcription factor NF-κB (33). NF-κB binds to the regulatory region of target genes that control the expression of MHC-II, CD80, and CD86 (34), and inhibition of NF-κB suppresses DC maturation and APC function (35, 36). Toll-like receptors (TLRs) and MyD88 are upstream regulatory factors of NF-κB (37) that relay the signal via MyD88 to activate NF-κB after stimulation (38). It has been reported previously that food-grade synthetic amorphous silica particles can directly initiate endosomal MyD88-dependent pathogen pattern recognition and signaling pathways in DCs (39). In this study, the expression of TLR4, TLR9, MyD88, and NF-κB mRNA and protein in DCs increased after direct exposure to silica, while they decreased after indirect silica exposure, compared with controls. These results indicated that silica exposure could influence the expressions of TLRs/MyD88/NF-κB in DCs. The data presented in this study demonstrate that silica exposure is a potent inhibitor of DC maturation. Thus, our results provide a possible explanation for the combined effects of silica on DC phenotype and function during NF-κB regulation dependent on the TLR/MyD88 pathway, resulting in a blockade of DC maturation. However, the direct involvement of TLR4, TLR9, MyD88, and NF-κB in DC responses to silica exposure needs to be researched in more detail.

Recent experimental and clinical studies suggest that persistent Th1/Th2 imbalance in the lung represents a possible additional mechanism involved in pulmonary fibrosis progression (15). Our recent investigations have demonstrated that DCs accumulated in lung tissues of rats exposed to silica dust and regulated the polarization of Th1/Th2 cells (40). In this study, the results showed that indirect silica exposure induces a blockade of DC maturation by reducing cell surface expression of these molecules, leading us to consider whether silica stimulation can alter the capacity of DCs to stimulate T cells. To determine whether DCs exposed to silica via direct or indirect mechanisms are capable of inducing T cell activation or tolerance, we analyzed the potential impact of silica on the ability of DCs to stimulate T cells. Cocultures of silica-exposed DCs and T cells demonstrated that silica-exposed DCs from both the DC+Silica and AM+Silica/DC groups could promote Th2 priming and IL-4 secretion (Th2 cytokine). Chronic silica exposure induces an imbalance in responder T cells in patients with silicosis (9). We have tested Th1/Th2 cells in the peripheral blood of silicosis patients with very similar results as displayed here (data not shown). These findings suggest that silica particles are not passively phagocytized but rather directly and indirectly alter the function of DCs to prime Th2 polarization of the adaptive immune response, and warrant further investigation.

The immune response relies on cell random migration to fulfill specific tasks (41, 42). DCs circulate in the blood and can migrate to peripheral tissues to take up antigens from infected, apoptotic, and necrotic cells (43). To investigate whether silica particles impact the migration and patrolling functions of DCs in pulmonary environments, we delivered CFSE-labeled DCs to silica-exposed and control groups of mice via tail vein injection. Our results showed that CFSE^+^ DCs were significantly increased in the lungs of silica-treated animals compared with controls after 8 days of exposure, suggesting that silica can affect DC migration and induce DC relocation to the pulmonary alveolae from the peripheral blood *in vivo*. Overall, the results presented in this study improve the understanding of key immunological mechanisms specific to the pathogenesis of silicosis that involve DCs.

## Ethics Statement

All animal procedures were approved by the ethics committee of Zhengzhou University (Henan, China), and performed in accordance with the Guiding Principles for the Care and Use of Laboratory Animals.

## Author Contributions

WY, SL, and CH conceived and designed the study. SL, LB, DZ, HZ, JH, DW, HC, and FF performed the experiments. SL performed the data analysis. SL, WY, and CH drafted the manuscript. FF reviewed the manuscript. All authors read and approved the final manuscript.

### Conflict of Interest Statement

The authors declare that the research was conducted in the absence of any commercial or financial relationships that could be construed as a potential conflict of interest.
