# Supplementary material for: Silica Particles Mediate Phenotypic and Functional Alteration of Dendritic Cells and Induce Th2 Cell Polarization
Source: Front Immunol. 2019 Apr 24;10:787. doi: 10.3389/fimmu.2019.00787 (PMC6491578; doi:10.3389/fimmu.2019.00787)
Supplement: Supplementary file 1 [file Data_Sheet_1.PDF]

Supplementary Material

Supplementary Figure 1

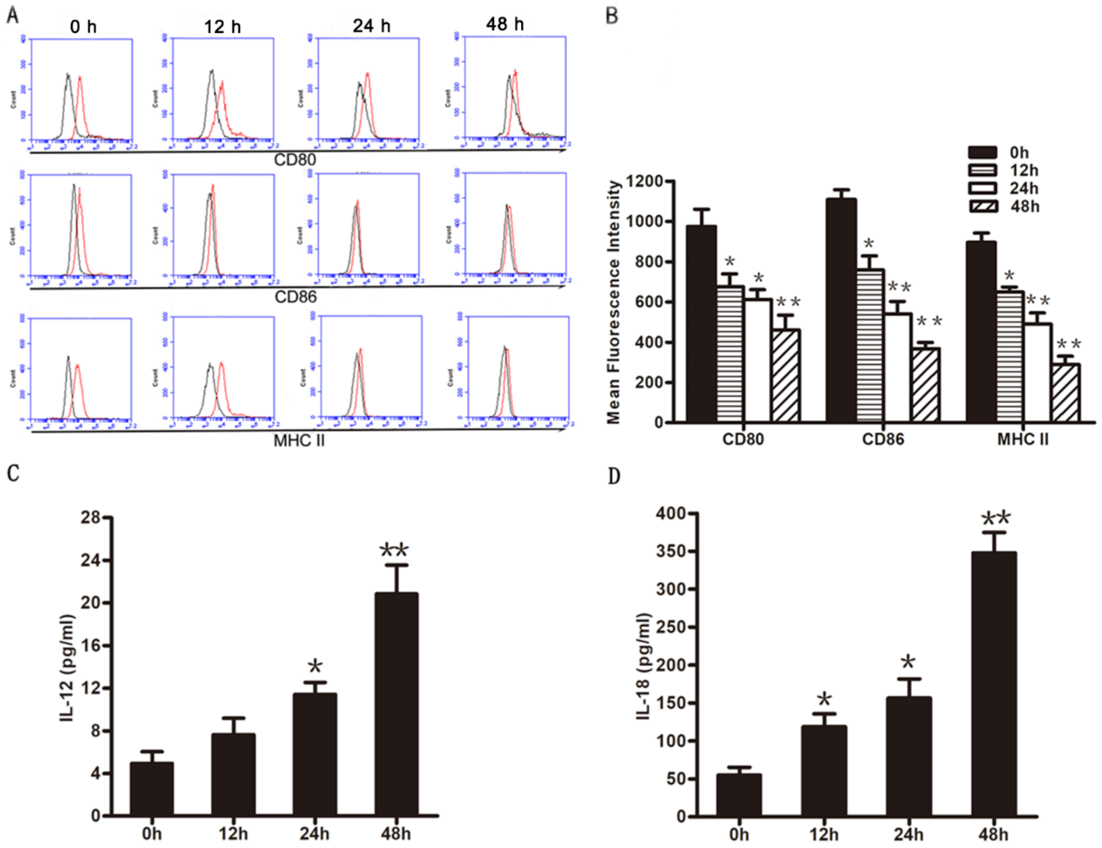

Supplementary Figure 1. Optimization of exposure time in experimental settings. DCs were treated with silica (80 µg/mL) for 12, 24, and 48 h, after which the functional activity of DCs was analyzed by measuring their expression of co-stimulatory molecules and cytokine production. Silica inhibited the expression of CD80, CD86 and MHC-II in DC and promoted the release of IL-12p70 and IL-18 in culture supernatants in a time dependent manner, with the highest inhibition and promotion detected at 48 h. 48 h of exposure was used for all functional analysis in this study.

Supplementary Figure 2

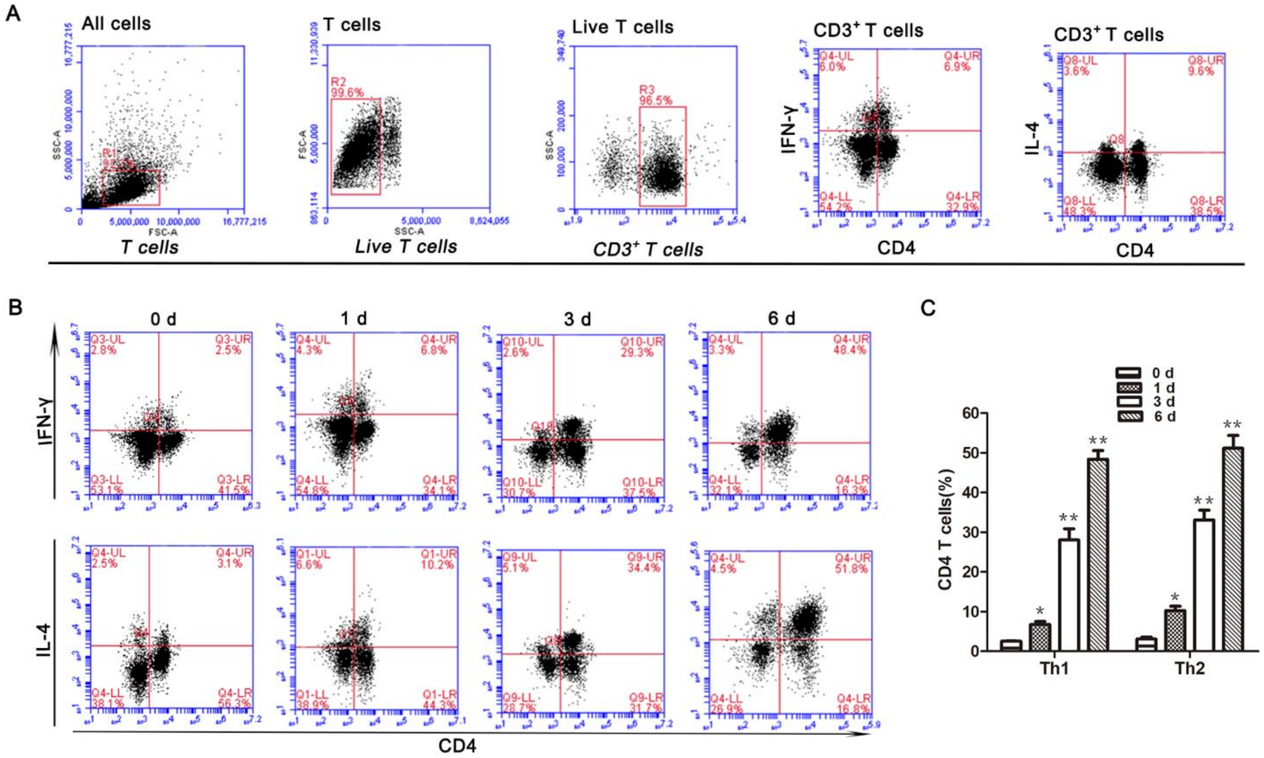

Supplementary Figure 2. The flow cytometry analysis of the positive control group. T cells were initially stimulated with plate-bound 3 µg/ml anti-CD3 and 2 µg/ml anti-CD28, and later with IL-12 (200 U/ml) and anti-IL-4 (10 µg/ml) for Th1, IL-4 (10µg/ml) for Th2. Culture was maintained for 0, 1, 3 and 6 d, after which proportions of Th1 and Th2 cells detected by flow cytometry (B). (A) Gating strategy for flow cytometry sorting of Th1 and Th2. (C) Data presented as a bar graph of the ratio of Th1 to Th2 cells of the positive control group.
